# Unlocking nerve regeneration: electrical stimulation and bioscaffolds to enhance peripheral nerve regeneration

**DOI:** 10.3389/fnins.2025.1594435

**Published:** 2025-05-16

**Authors:** Vanshit Thakkar, Melod Mehdipour, Stephano Chang

**Affiliations:** ^1^Department of Neurobiology, Physiology, and Behavior Science, University of California, Davis, Davis, CA, United States; ^2^California Northstate University, College of Medicine, Elk Grove, CA, United States; ^3^Department of Neurological Surgery, University of California, Davis Medical Center, Sacramento, CA, United States

**Keywords:** peripheral nerve injury, nerve regeneration, peripheral nerve regeneration/repair, Schwann cell reprogramming, electrical stimulation, natural and synthetic scaffolds, bioengineered nerve conduits, machine learning bioprinting optimization

## Abstract

Peripheral nerve injury (PNI) is a challenging clinical problem resulting in disabling sensorimotor deficits, which may become permanent if recovery does not take place in a timely manner. In this review, we examine recent insights into key molecular mechanisms—particularly MAPK/ERK and PI3K/Akt—that govern Wallerian degeneration, Schwann cell (SC) reprogramming, and macrophage polarization. These and other critical steps in the axonal regeneration process must be understood and navigated for a therapeutic approach to be successful. We highlight emerging therapeutic strategies, such as electrical stimulation (ES), which appears to work by activating many of these pro-regenerative gene networks, both in neurons and non-neuronal support cells. Advances in biomaterial engineering, including natural and synthetic scaffolds enriched with growth factors, also show promise in facilitating axonal regeneration across nerve gaps. We postulate that integrating optimized ES protocols with innovative scaffold designs will allow for synergies to further enhance axonal regeneration and functional recovery.

## Introduction

Peripheral nerve injury (PNI) has an incidence between 10 and 20 per 100,000 annually in the US (Tapp et al., 2019) and can cause lasting sensorimotor deficits, pain, and diminished quality of life (Wojtkiewicz et al., 2015). While peripheral nerves can regenerate, fewer than half of patients with severe PNIs achieve satisfactory recovery after surgical repair (Gordon and Gordon, 2010). Prolonged muscle denervation results in irreversible fibrofatty infiltration, and thus treatment delays and long regeneration distances often lead to poor outcomes (Fu and Gordon, 1997; Gordon, 2016; Wojtkiewicz et al., 2015). There is a critical need to develop strategies to accelerate the slow, intrinsic growth rate of axons, and to effectively bridge nerve gaps to facilitate end-organ reinnervation. In this narrative review, we review the mechanistic underpinnings of PNI and regeneration, as well as recent advances and future directions in enhancing nerve regeneration and functional recovery, focusing on electrical stimulation and scaffolds.

### Structural classification of peripheral nerve injuries

**Figure 1:**
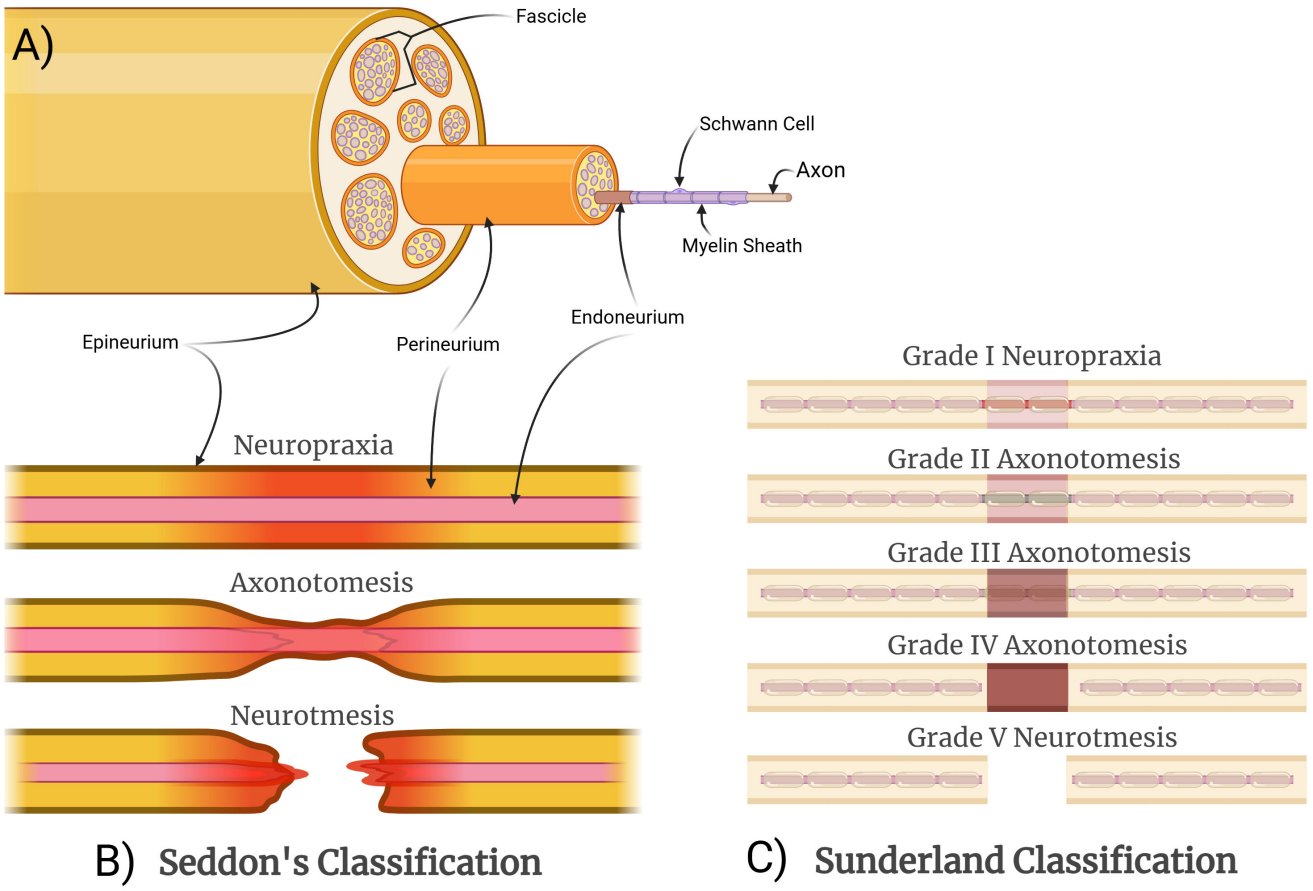
Structural organization and classification of peripheral nerve injuries. **(A)** A schematic cross section of a nerve, with fascicles and individual axons demonstrated for illustrative purposes. **(B)** Seddon categorized nerve injuries based on the structural components involved and the resulting functional disturbances as well as the potential for recovery. Neuropraxia is the mildest injury, resulting from demyelination only without axonal disruption, causing a temporary conduction block. Axonotmesis involves axonal injury but with preservation of surrounding structures, allowing for a slow but spontaneous recovery. Neurotmesis involves complete injury of the nerve, requiring surgical intervention. **(C)** Sunderland expanded theoretically on this concept by differentiating between different severities of axonotmesis: Grade II is the mildest with intact endoneurial tubes; Grade III involves disruption of endoneurial tubes but not the perineurium; and Grade IV involves disruption of the endoneurium and the perineurium.

Peripheral nerves are intricately organized structures that efficiently transmit signals (Dong Y. et al., 2023) while accommodating significant bodily movements with elastic stretch (Dong X. et al., 2023). These nerves are composed of axonal bundles supported by Schwann cells (SCs) which play a pivotal role in the myelination and metabolic support of the axons (Gordon and Gordon, 2010; Izhiman and Esfandiari, 2024; Lopes et al., 2022). Three layers of connective tissues encapsulate these bundles of axons. The innermost layer is the endoneurium, enveloping the individual nerve fibers and providing an optimal microenvironment for axonal function. The perineurium protects nerve fascicles, which are bundles of axons within the nerve. The epineurium encapsulates the entire nerve, provides structural support, encloses the nerve's vascular supply, and protects it from external mechanical tension and chemical effects in the event of injury (Figure 1A; Griffin et al., 2014; Lopes et al., 2022; Mahan, 2020; Ni et al., 2023; Sunderland, 1951). Epineurium facilitates physical nerve movement within tissues (Lopes et al., 2022). Peripheral nerves transmit bidirectional signals between the central nervous system (CNS) and the peripheral tissues. The efferent fibers in the peripheral nerves carry motor commands from the CNS to the target muscles innervated by the nerves (Izhiman and Esfandiari, 2024; Lopes et al., 2022; Zhao et al., 2024). In contrast, the afferent fibers communicate sensory information from the target periphery back to the CNS (Zhao et al., 2024). The efferent and afferent nerve fibers consist of axons that could either be myelinated or unmyelinated (Fu and Gordon, 1997; Loo et al., 2015; Pfister et al., 2007). Despite the unmyelinated axons being slower in communication, they are crucial for autonomic and pain transmission (Pfister et al., 2007).

Peripheral nerve injuries are classified by anatomical location (e.g., distal ulnar nerve, proximal upper trunk of brachial plexus), as well as by severity. The classical grading system for injury severity that is still widely used and referenced today was formulated by Seddon and included neurapraxia, axonotmesis, and neurotmesis, based on the structural components of the nerve that were injured (Figure 1B). Neurapraxia is an injury without damage to the axons that causes partial nerve interruptions. Axonotmesis is axonal damage with intact connective sheaths that decrease the gap between the damaged axons. Neurotmesis is the most severe form of nerve injury and involves the complete disruption of axon, myelin sheath, and all connective tissue layers. Spontaneous recovery of the damaged nerve at the neurotmesis level is poor because the structural framework of the nerve is lost and surgical intervention is typically required (Seddon, 1943). Sunderland later expanded on this concept by differentiating between severities of axonotmesis (Figure 1C). Grade I describes localized demyelination, previously classified as neurapraxia by Seddon. Grade II injuries result in axonal loss and intact endoneurium. Grade III injuries consist of damaged axons and endoneurium while leaving the perineurium intact. Grade IV injuries are represented by damaged axon, endoneurium, and perineurium, with intact epineurium. Grades II–IV can be considered variants of axonotmesis and neurotmesis. Grade V injuries, also referred to as neurotmesis by Seddon, represented the most extreme case of a complete disruption of the nerve (Sunderland, 1951). Both classifications maintain that progressive damage to axons and their connective tissue sheaths is associated with poorer clinical outcomes (Sunderland, 1951). Mackinnon later added a sixth grade for nerve injuries, which can be any combination of multiple Sunderland's injury grades (I–V) within the same nerve and likely better reflects clinical scenarios, particularly crush injuries, where optimal surgical interventions may differ by fascicle (Mackinnon, 1989).

The prognosis of PNI is also determined by the injury location, generally being classified as proximal or distal. Proximal injuries pose greater challenges since axons must traverse longer gap distances to reach their targets. These injuries are associated with poorer outcomes because of the appreciable risk of axon misdirection (Al-Majed et al., 2000a; Fu and Gordon, 1997; Sunderland, 1951). A common source of misrouting is due to nerve bifurcation that results in functional mismatches between motor and sensory neurons. Inadequate restructuring of extracellular matrix (ECM) and insufficient SC guidance compounds this problem. Extended regeneration time also weakens motor and sensory preference for reinnervation (Al-Majed et al., 2000a; Gordon, 2016). Longer gap distances lead to a decline of SC integrity in the regenerative microenvironment. Extended recovery times cause SCs to lose their repair capabilities, reducing neurotrophic factor secretion, and allowing fibrotic processes to disrupt ECM formation during axonal growth (Chu et al., 2022; Fu and Gordon, 1997; Gordon, 2016; Hardy et al., 2024). In contrast, distal injuries are associated with shorter axonal gap distances, which may lead to improved outcomes. Successful regeneration, on the other hand, still relies on efficient communication between SCs and immune cells (Chu et al., 2022; Fu and Gordon, 1997; Wan et al., 2025).

### Molecular and pathophysiological changes in PNI

**Figure 2:**
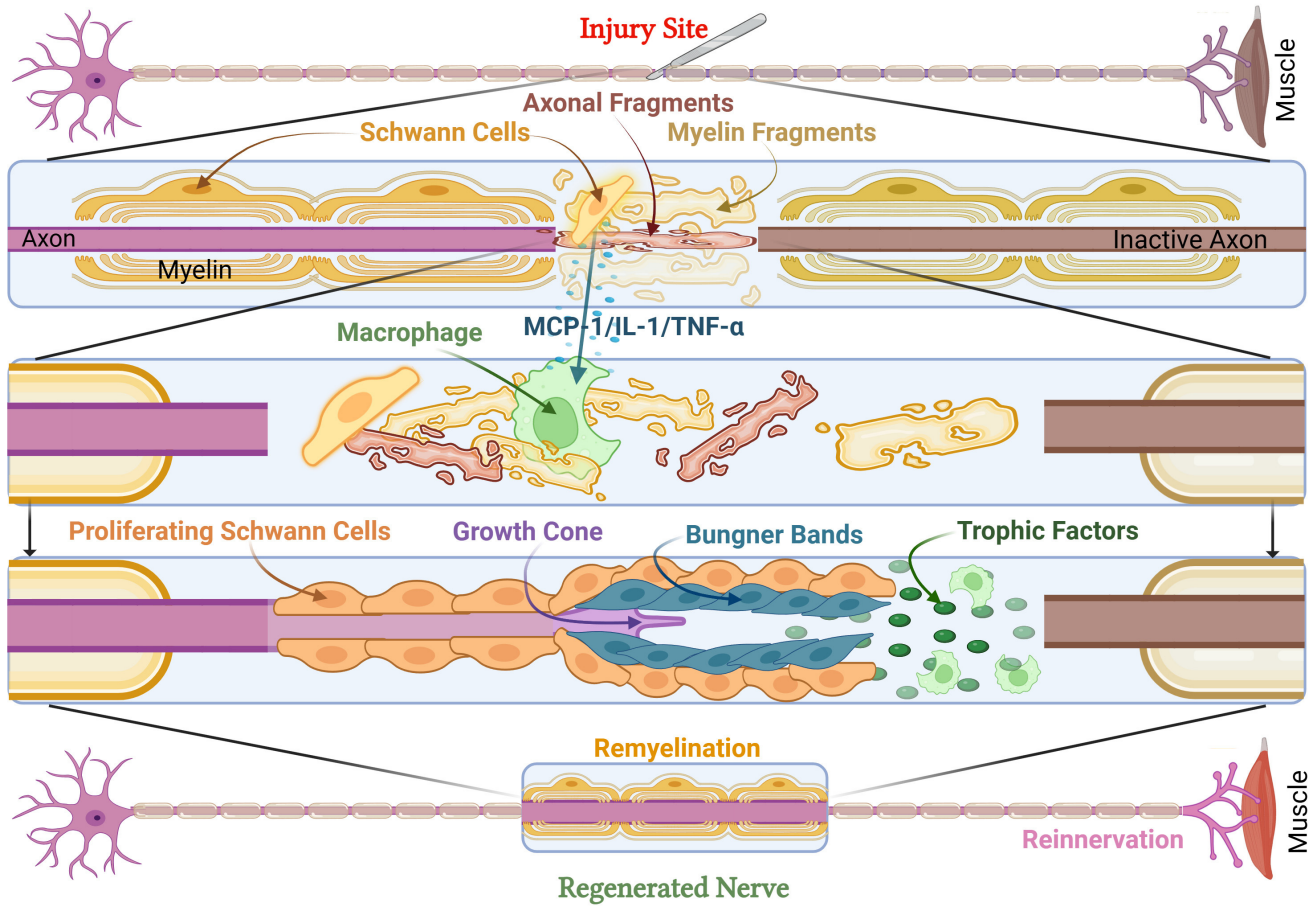
Cellular and molecular responses to peripheral nerve injury. Peripheral nerve injury leads to muscle denervation. At the injury site, axonal and myelin fragments form debris, while damaged SCs release MCP-1, IL-1, and TNF- α to activate macrophages and other SCs to clear debris. This leads to an increase in trophic factors and the formation of Büngner Bands with proliferating SCs to support the growth cone. This results in remyelinated and regenerated nerves as well as reinnervated muscle. MCP-1, Monocyte Chemoattractant Protein-1; IL-1, Interleukin-1; TNF-α, Tumor Necrosis Factor-alpha.

Following axonotmesis, the disruption of axonal membranes triggers a rapid influx of calcium ions, activating proteolytic enzymes, including calpains, that degrade cytoskeletal structures (Izhiman and Esfandiari, 2024; Lopes et al., 2022; Zhao et al., 2024). This disrupts axonal transport, preventing the delivery of essential nutrients and organelles from the soma to the distal axon, ultimately leading to the degeneration of the distal axonal segment. This process, eponymously termed Wallerian degeneration, after British neurophysiologist Augustus Waller, is an essential part of PNI (Waller, 1851). While axonal fragmentation initially generates debris that can impede regeneration, it is a necessary step in promoting nerve repair, as injured axons and myelin debris must be eliminated before axonal regeneration can take place (Figure 2; Izhiman and Esfandiari, 2024; Lopes et al., 2022; Zhao et al., 2024). Delays in Wallerian degeneration may lead to reduced regeneration rates and incomplete recovery, especially sensory axons. A genetic study of C57BL/Ola (delayed Wallerian degeneration) mice discusses increased axonal degeneration, debris accumulation, and lowered SC association with axons (Brown et al., 1992). This can result in long-term deficits and neuropathic pain. This is highlighted in WldS (Wallerian degeneration slow) mutant mice, which demonstrate delayed Wallerian degeneration, but also demonstrate delayed axonal regeneration as a consequence (Brown et al., 1994; Coleman and Freeman, 2010).

Schwann cells play a pivotal role in the process of Wallerian degeneration, dedifferentiating, and actively degrading the myelin sheath, and secreting monocyte chemoattractant protein-1 (MCP-1), which recruits macrophages to the injury site (Figure 2). These macrophages phagocytose myelin debris, creating an environment permissive for axonal regeneration. In addition, SCs undergo transcriptional reprogramming, downregulating genes associated with myelin production (e.g., structural protein zero and myelin basic protein) while simultaneously upregulating repair-associated genes (Chen et al., 2007; Izhiman and Esfandiari, 2024; Jessen and Mirsky, 2016; Morgan et al., 1994). This transition to SC repair phenotype enables the axons to align longitudinally by repair-phenotype SCs forming scaffold known as the Band of Büngner (Hardy et al., 2024; Izhiman and Esfandiari, 2024; Wan et al., 2025). SCs also secrete neurotrophic factors such as brain-derived neurotrophic factor (BDNF) and nerve growth factor (NGF), which promote axonal elongation and survival (Chen et al., 2024; Chu et al., 2022; Hardy et al., 2024; Izhiman and Esfandiari, 2024). Thus, Wallerian degeneration is a tightly regulated process that is essential for successful axonal regeneration. The injured nerve is ultimately primed by the breakdown of damaged axons, macrophage-mediated debris clearance, and the establishment of a pro-regenerative microenvironment. Additionally, exosomes play a pivotal role in regeneration (Ching and Kingham, 2015; Lai and Breakefield, 2012). An *in-vitro* study has indicated that SC-derived exosomes positively affect axonal regeneration after crush injuries (Lopez-Verrilli et al., 2013).

The inflammatory responses in PNI occur in two distinct phases, which are tightly regulated to ensure optimal nerve regeneration. In the pro-inflammatory phase, cytokines such as tumor necrosis factor-alpha (TNF-α) and interleukin-1 beta (IL-1β), recruit pro-inflammatory (M1) macrophages to clear debris efficiently (Chu et al., 2022; Dinescu et al., 2025; Hardy et al., 2024). However, prolonged stimulation from these pro-inflammatory mediators may aggravate pain and tissue damage (Chen et al., 2024). To counteract this, transforming growth factor-beta (TGF-β) combined with platelet-derived growth factor (PDGF) recruit macrophages to the injury site (Fu and Gordon, 1997) and promote M1-to-M2 (pro-repair) macrophage transition (Wan et al., 2025). M2 macrophages release anti-inflammatory interleukin-10 (IL-10) and cascade axonal repair (Hardy et al., 2024). TGF-β further induces SC proliferation and modulates neurotrophic factor synthesis, which play a major role in nerve regeneration (Fu and Gordon, 1997). When administered with forskolin, TGF-β1 demonstrates substantial improvements in axonal regeneration by supporting SC proliferation, decreasing apoptotic signaling, extending, and reactivating SCs to promote survival, and supporting axonal sprouting (Sulaiman and Nguyen, 2016). Several molecular pathways, including MAPK/ERK and PI3K/Akt, regulate this transition, facilitating growth cone formation, cytoskeletal reorganization, and neurotrophic factor secretion (Chu et al., 2022; Hardy et al., 2024).

Furthermore, nerve growth factor (NGF), glial cell line-derived neurotrophic factor (GDNF) and BDNF aid in ECM remodeling and the formation of the Bands of Büngner (Hardy et al., 2024; Izhiman and Esfandiari, 2024; Liu et al., 2025; Lopes et al., 2022). This is facilitated by the extracellular signal-regulated kinase (ERK), c-Jun n-terminal kinase (JNK), mitogen-activated protein kinase (MAPK), and phosphoinositide 3-kinase/protein kinase B (PI3K/AKT) pathways (Chu et al., 2022; Hardy et al., 2024; Zhao et al., 2024). JNK signaling contributes to axonal elongation by enhancing actin filament assembly. In addition, stress signals activate the p38 component of MAPK to regulate the inflammatory response by producing neurotrophin. ERK and p38 MAPK play crucial roles in the M1-to-M2 macrophage transition, driving IL-10 and VEGF production during the resolution of inflammation (Izhiman and Esfandiari, 2024; Zhao et al., 2024).

**Figure 3:**
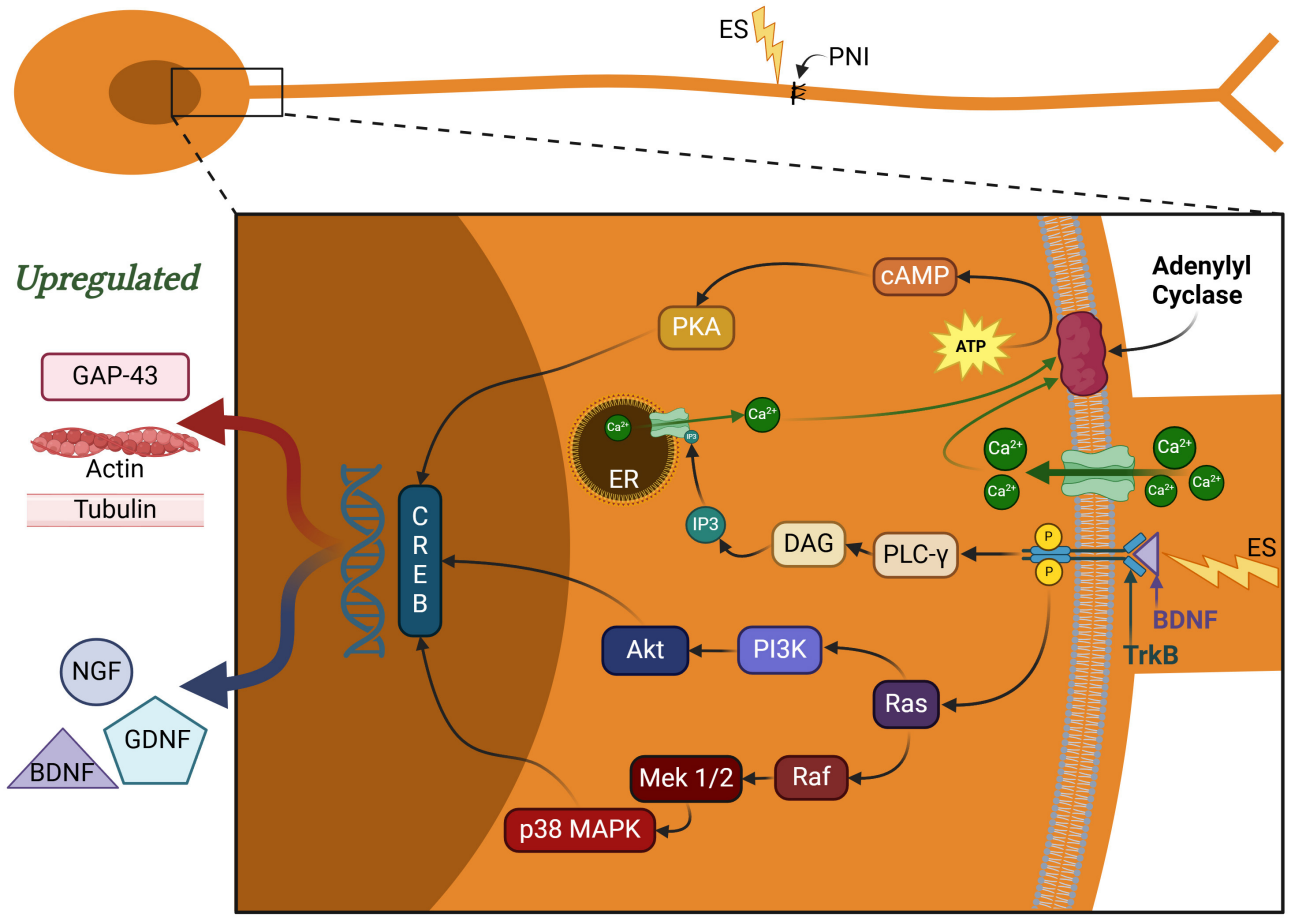
Signaling pathways regulating nerve regeneration. These pathways are activated by brain-derived neurotrophic factor (BDNF) binding to tropomyosin kinase receptor B (trkB). MAPK and PI3K pathways play a critical role in CREB activation, which aids in synaptic plasticity, neuronal remodeling, and pain receptor upregulation. Additional functions of CREB include neuronal excitability, pain amplification, and SC proliferation and dedifferentiation. PLC-γ pathway aids in additional calcium release from endoplasmic reticulum (ER). The calcium influx and interaction with adenylyl cyclase aids in cAMP production, which further contributes to the CREB activation. BDNF, brain-derived neurotrophic factor; NGF, nerve growth factor; CREB, cAMP response element binding protein; cAMP, cyclic adenosine monophosphate; RAGs, regeneration-associated genes; MAPK, mitogen-activated protein kinase; PI3K, phosphoinositide 3-kinase; PLC-γ, phospholipase C gamma; DAG, diacylglyerol; IP_3_, inositol 1,4,5 triphosphate; PKA, protein kinase A; ATP, adenosine triphosphate; ER, endoplasmic reticulum; ES, electrical stimulation; PNI, peripheral nerve injury; GAP-43, growth-associated protein 43; GDNF, glial cell-derived neurotrophic factor.

BDNF and NGF bind to their respective tropomyosin receptor kinase B and A (TrkB and TrkA) which support the PI3K signaling pathway in different ways (Harrington and Ginty, 2013; Sharma et al., 2010). NGF-TrkA interaction has shown to be crucial for neuronal support and blocking apoptotic signaling through PI3K pathway (Harrington and Ginty, 2013; Sharma et al., 2010). Additionally, BDNF-TrkB interaction also leads to the generation of phosphatidylinositol-3,4,5-triphosphate (PIP3; McGregor and English, 2019), further supporting the PI3K pathway (Figure 3). PIP3 recruits and activates protein kinase B (AKT), which induces mTOR and forkhead box O (FOXO) transcription factors (Vanhaesebroeck et al., 2012). The activation of mTOR promotes growth cone advancement and axonal repair by the enhancement of protein synthesis. AKT also induces VEGF, thereby enhancing angiogenesis that is essential for axonal regeneration (Izhiman and Esfandiari, 2024; Loo et al., 2015).

Beyond regeneration, the PI3K/AKT pathway drives macrophage polarization to the M2 phenotype by increasing IL-10 secretion while suppressing pro-inflammatory NF-kB activity (Chen et al., 2024; Izhiman and Esfandiari, 2024; Zhao et al., 2024). PI3K/AKT signaling also maintains the survival of SCs under oxidative stress during inflammation, myelin clearance, SC migration, and axonal alignment during regeneration (Chu et al., 2022).

## Therapeutic interventions

Severe PNI (Grade V Neurotmesis) often leads to permanent functional impairment since spontaneous regeneration relies on the presence of intact connective tissue sheaths guiding axonal regeneration. If the injury is proximal, prolonged muscle denervation can result in irreversible fibro-fatty infiltration and loss of function. In these circumstances, surgical interventions can improve the likelihood of favorable outcomes. Surgical interventions classically include microsurgical repair, nerve grafting, and nerve transfers, though recent advances show promise for electrical stimulation and the implantation of scaffolds (Costello et al., 2023; Hardy et al., 2024; Juckett et al., 2022). Here, we discuss recent advances and future directions for electrical stimulation as well as the use of scaffolds to enhance peripheral nerve regeneration in neurotmesis injuries.

### Direct electrical stimulation (ES) at injury site

Internal electrical stimulation (ES) of the proximal nerve segment is a technique in which a brief duration of electrical currents is directly delivered to injured nerves to promote the speed and accuracy of axonal regeneration (Al-Majed et al., 2000a; Ghosh-Roy et al., 2010). It has shown promise in both animal studies and pilot clinical studies (Al-Majed et al., 2000a; Gordon et al., 2010; Power et al., 2020; Sayanagi et al., 2021; Wong et al., 2015; Zhang et al., 2023), through the activation of pro-regenerative gene networks, secretion of neurotrophic factors, and inhibition of inflammation (Chen et al., 2024; Chu et al., 2022; Costello et al., 2023; Hardy et al., 2024; Juckett et al., 2022; Ni et al., 2023; Ong Sio et al., 2023). Internal ES is more invasive than external ES (applied superficially), and potentially carries more clinical risks associated with additional surgery and electrode placement on the nerve (Chu et al., 2022).

#### Molecular mechanisms of direct ES

The direct nerve ES method consists of placing small electrodes directly on the exposed nerves post-anastomosis. A moist environment must be maintained with warm saline to prevent nerve desiccation (Al-Majed et al., 2000a,b; Brushart et al., 2002). This is to ensure stable electrode connection to the nerve once the electrode has been placed. The type of electrode, wires, or needles that are used vary depending on the clinical scenario. Cuff electrodes are usually made of pliable silicone or polytetrafluoroethylene (PTFE). They can be placed around the repaired nerve segment and loosely secured with a suture or wound clip (Birenbaum et al., 2023; Koo et al., 2018; Park et al., 2019). Alternatively, wire or needle electrodes that are made of two fine stainless steel or platinum-iridium wires can be positioned on or under the epineurium to serve as the cathode, while the other is placed on adjacent tissue as the anode (Raslan et al., 2019; Sayanagi et al., 2021). When electrodes are placed directly on the repaired nerve, sensory, and motor axons in the nerve trunk are depolarized by ES. The electrical currents are focused directly on the axons since the nerve fascicles are exposed and isolated from surrounding soft tissue. This configuration minimizes dissipation of electrical charge through the subcutaneous layer (Al-Majed et al., 2000a; Raslan et al., 2019). Action potentials propagate orthodromically and antidromically upon electrode activation. The resulting synchronized depolarization induces growth-associated genes and neurotrophic factors (Al-Majed et al., 2000a; Geremia et al., 2007; Huang et al., 2013; Sayanagi et al., 2021). Uniform axonal depolarization within a short intraoperative window minimizes mismatches which enhances the accuracy of motor axons reconnecting with motor fascicles. Staggered regeneration is also minimized, ultimately improving functional outcomes (Al-Majed et al., 2000a,b; Brushart et al., 2002; Costello et al., 2023; Geremia et al., 2007; Juckett et al., 2022; McGregor and English, 2019). Retrograde action potentials in the neuronal soma will lead to increased calcium influx, thereby activating the MAPK/ERK, PI3K/Akt, and cAMP pathways in the motor and sensory neurons (Al-Majed et al., 2000b; Costello et al., 2023; Ghosh-Roy et al., 2010; Hardy et al., 2024; McGregor and English, 2019).

The calcium influx triggers the release of mature BDNF (mBDNF) stored in neuronal vesicles. Additionally, ES results in the rapid upregulation of the mRNA expression of both BDNF and its cognate receptor, trkB (McGregor and English, 2019). Overall, this has the effect of inducing the BDNF-trkB signaling pathway. When mBDNF binds to trkB, it initiates receptor activation through dimerization and subsequent autophosphorylation of intracellular tyrosine residues. This activates three primary intracellular signaling pathways: phospholipase C gamma (PLC-γ), PI3K, and MAPK/ERK (Figure 3; McGregor and English, 2019). The MAPK/ERK pathway phosphorylates microtubule-associated proteins, supporting cytoskeletal remodeling (Chu et al., 2022; Izhiman and Esfandiari, 2024; Juckett et al., 2022). ERK specifically facilitates the synthesis and delivery of tubulin and actin-like cytoskeletal components to the growth cone. Increased PI3K signaling activity promotes actin polymerization, microtubule stabilization, and the inhibition of pro-apoptotic factors (Hardy et al., 2024; Ni et al., 2023). Actin filament remodeling is highly dynamic and concentrated at the growth cone. PI3K signaling upregulates filipodia and lamellipodia production to enhance the motility of regenerating axons to their respective targets (Hardy et al., 2024; Izhiman and Esfandiari, 2024; Ni et al., 2023). The inhibition of pro-apoptotic signals strengthens cytoskeletal integrity by preventing growth cone collapse (Chu et al., 2022; Izhiman and Esfandiari, 2024). Thus, the MAPK, PI3K, and PCL-γ pathways play a crucial role in promoting the transcription of these so-called regeneration-associated genes (RAGs) and subsequent axonal regeneration (Chu et al., 2022; Juckett et al., 2022).

ES likely improves nerve regeneration by accelerating and enhancing this natural process, with gradual elevation in BDNF and trkB mRNA expression by 2-fold at 7 days post PNI without ES (Al-Majed et al., 2000b). In contrast, ES induced this same upregulation in mRNA expression within 8 h of injury, with further increases at 2 days post PNI (Geremia et al., 2007). Furthermore, the process is initiated across all injured axons in the nerve simultaneously with ES, rather than in the temporally staggered manner seen with natural PNI. This manifests as an accelerated and synchronized neurite outgrowth across the injury site, when compared to PNI without ES (Brushart et al., 2002).

**Figure 4:**
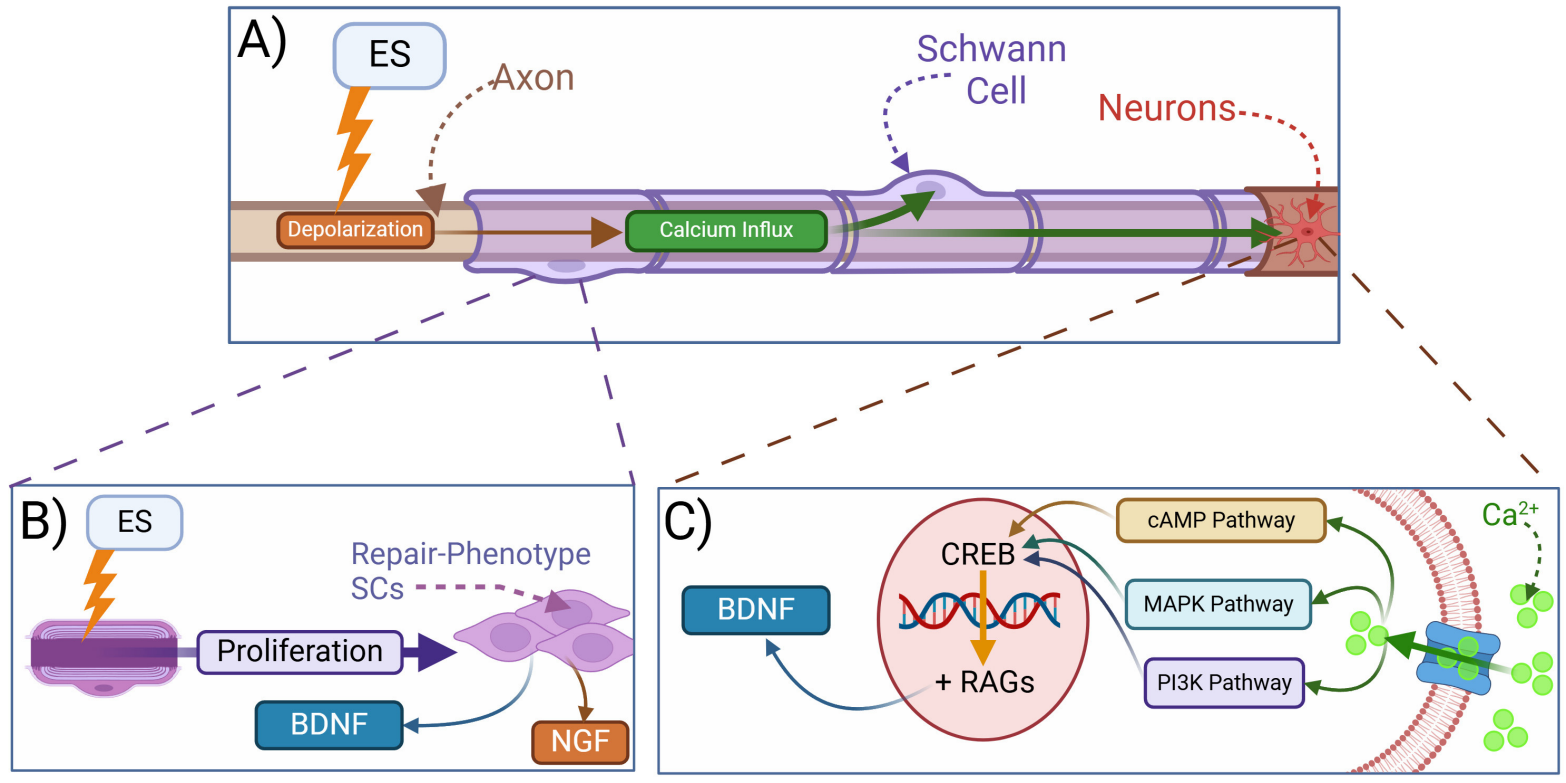
Mechanisms of electrical stimulation in peripheral nerve regeneration. **(A)** Electrical stimulation (ES) leads to depolarization and intracellular calcium influx. **(B)** ES promotes the reprogramming of SCs into a repair phenotype, which secrete neurotrophins and support axonal regeneration. **(C)** Mechanistically, ES depolarization of injured axons leads to the activation of calcium-dependent signaling pathways in neurons resulting in the release of BDNF, among other neurotrophic factors, as well as the transcription of regeneration-associated genes (RAGs). BDNF, brain-derived neurotrophic factor; NGF, nerve growth factor; CREB, cAMP response element binding protein; cAMP, cyclic adenosine monophosphate; RAGs, regeneration-associated genes; MAPK, mitogen-activated protein kinase; PI3K, phosphoinositide 3-kinase.

ES also increases calcium influx in SCs (Figure 4A) and promotes their proliferation and transition toward a pro-repair phenotype (Hu et al., 2019; Izhiman and Esfandiari, 2024). ES-stimulated SCs secrete more neurotrophic factors such as BDNF and NGF, potentially contributing to neuronal survival and axonal outgrowth (Figures 4B, C; Al-Majed et al., 2000b; Du et al., 2018; Huang et al., 2013; Izhiman and Esfandiari, 2024; Ong Sio et al., 2023; Song et al., 2021). ES also modulates local SCs to increase the release of exosomes that lead to a shift in the macrophage phenotype from M1 (pro-inflammatory) to M2 (pro-repair; Hardy et al., 2024; McLean and Verge, 2016; Ni et al., 2023; Zhao et al., 2024). This ultimately downregulates pro-inflammatory cytokines and promotes pro-repair factors to expedite myelin debris clearance and remyelination to prime the microenvironment for optimal functional recovery (Chu et al., 2022; Izhiman and Esfandiari, 2024; Keane et al., 2022; McLean and Verge, 2016; Ni et al., 2023; Zhao et al., 2024).

#### Optimal direct ES parameters in clinical setting

Several rodent PNI studies primarily utilized ES protocols that last 1 h in length immediately after nerve repair surgery. These studies consistently demonstrated enhanced nerve conduction velocity, increased axon counts, and earlier reinnervation when compared to control groups (Al-Majed et al., 2000a; Brushart et al., 2002). This led to the presumption that prolonged depolarization could exert maximal effect, which was shown false when prolonged ES yielded poor outcomes (Al-Majed et al., 2000a; Geremia et al., 2007). The physiological firing range of most peripheral motor neurons lie between 5 and 30 Hz, making 20 Hz a clinically relevant frequency for ES to enhance neuronal conduction (Al-Majed et al., 2000b; Chu et al., 2022; Geremia et al., 2007; Senger et al., 2018; Wang et al., 2024). This frequency optimally balances nerve excitation while minimizing the risk of conduction blocks that occasionally occur at elevated frequencies (Du et al., 2018; Wan et al., 2010; Wang et al., 2024). Several studies suggest that lower frequency ES can prevent additional damage and yield optimal growth results (Izhiman and Esfandiari, 2024; Lu et al., 2008; Zuo et al., 2020). In their seminal work, Al-Majed et al. (2000a) selected a stimulation frequency of 20 Hz based on the mean physiological frequency of motor neuron discharge, and tested several durations of stimulation, including 1 h, 1 day, 1 week, and 2 weeks, finding that just 1 h of 20 Hz ES significantly enhanced axonal regeneration and functional recovery. Further, with a rigorous and systematic interrogation of stimulation parameters in an *in vitro* optogenetic study seeming to substantiate the 20 Hz, 1 h protocol (Park et al., 2015). Therefore, the 1-h at 20 Hz protocol became widely regarded as the gold standard based on the premise that sustained action potentials promote uniform axonal sprouting and sufficient calcium influx (Costello et al., 2023; Gordon et al., 2010; Hardy et al., 2024; Izhiman and Esfandiari, 2024; Juckett et al., 2022; Ni et al., 2023; Park et al., 2015; Power et al., 2020; Wong et al., 2015; Zhao et al., 2024).

However, several rodent studies (Calvey et al., 2015; Roh et al., 2022; Sayanagi et al., 2021) and a recent clinical study (Zhang et al., 2023) have suggested that shorter stimulation durations of 10–15 min may be sufficient to trigger a similar response, while greatly improving the feasibility of ES as an intraoperative therapy. In optically stimulated neurons, neurite outgrowth drastically increased in 15–45-min durations, plateaued after 45 min stimulation, reached maximum growth peak at 1 h, and had diminishing results for 3 h to 3-day durations (Park et al., 2015). Extended stimulation beyond the point of signaling cascade saturation appears to be unnecessary. Rat sciatic nerve models compared 15-min and 1-h ES durations and found no significant differences in final muscle force or compound muscle action potentials at multiple timepoints (Roh et al., 2022). Similar studies utilizing 10- or 20- min perioperative ES protocols showed that conduction velocity and axonal growth were comparable to the 1-h approach (Costello et al., 2023; Ni et al., 2023). Both (10- and 60-min) stimulation durations present similar results in increased RAG expression (Sayanagi et al., 2021), which shows shorter durations could be increasingly favored in clinical settings. Quicker stimulation times may spare clinical resources without compromising patient outcomes (Ni et al., 2023) and potentially reduce risk from prolonged surgical duration.

ES applied immediately post-PNI has shown to double the number of regenerating sensory neurons in femoral nerves (Geremia et al., 2007). ES potentially mimics the retrograde calcium wave (McGregor and English, 2019), initially triggered at PNI to enhance axonal growth and nerve regeneration (Mar et al., 2014). Immediate ES can accelerate axonal crossing, and reinnervate motoneurons to the targets earlier compared to delayed regeneration and poor outcomes in non-stimulated nerves (Al-Majed et al., 2000a; Brushart et al., 2002; McGregor and English, 2019). Brief low-frequency acute ES following PNI repair can significantly enhance motor nerve regeneration, remyelination, and muscle reinnervation compared to delayed stimulation protocols (Geremia et al., 2007; Nicolas et al., 2018; Wan et al., 2010), and potentially reduce the staggered regeneration delay in axons navigating the gap (Al-Majed et al., 2000a; Huang et al., 2013; Senger et al., 2018). ES can also accelerate myelin debris clearance (Li et al., 2023), which suggests that earlier implementation of ES could be linked to better regenerative outcomes and functional recovery. However, delayed ES after 4–24 weeks can drastically enhance axonal regeneration, remyelination, upregulate BDNF expression, and improve motor functional recovery (Huang et al., 2013). Thus, acute stimulation post-PNI is most optimal for ensuring proper reinnervation and regeneration (Nicolas et al., 2018), but if not possible, delayed stimulation can still be clinically relevant to improve prognosis without stimulation (Huang et al., 2013).

Whether or not repeating these stimulations at regular intervals might further enhance the therapy remains an open question, with Koo et al. (2018) reporting that rodents undergoing 6 days of 20 Hz ES for 1 h per day had better functional recovery after PNI than rodents receiving 3 days or 1 day of 1 h per day ES, while Park et al. (2019) found no difference between a single application of ES compared to a 2-week regimen of ES applied for 1 h every third day. Interestingly, several studies potentially highlight the diminishing effects of ES as a crucial limitation (Mendez et al., 2016; Raslan et al., 2019). Mendez et al. (2016) found a statistically significant enhancement in facial nerve performance (whisking amplitude) in the brief ES group at week 2 compared to the control group, but noticed the difference not being significant at weeks 4 and 6. This suggests that ES, in facial nerves, led to an initial surge in regenerative signaling till 2 weeks but diminished in effectiveness after. Similarly, Raslan et al. (2019) showed no measurable long-term improvements in function (video analysis of whisking motion) or precision of reinnervation after brief ES in facial nerve injury. Which poses the question, could repetitive brief ES once every 2–4 weeks, instead of every third day (Park et al., 2019), improve the prognosis in cases with diminishing effects of ES after a few weeks? Conversely, Raslan et al. (2019) noticed enhanced functional recovery and significantly improved preferential motor reinnervation (PMR) when brief ES was applied to femoral nerve. Hence, brief low-frequency ES can still be beneficial (Al-Majed et al., 2000a; Geremia et al., 2007; Sayanagi et al., 2021), but the mechanistic response could depend on the nerve type (Ni et al., 2023; Raslan et al., 2019), potentially due to ES affecting sensory-motor specificity (McGregor and English, 2019) and enhancing sensory neuron regeneration (Geremia et al., 2007; Raslan et al., 2019), which could affect motor axon outgrowth and synchronized regeneration (Brushart et al., 2002; McGregor and English, 2019; Raslan et al., 2019). A more thorough interrogation of stimulation protocols and regimens is important to guide clinical translation. For example, it is not known if further spaced repetitions of ES (e.g., once a week, once a month) might enhance axonal recovery by re-boosting the release of neurotrophic factors and the transcription of pro-regenerative genes at more appropriate time scales since we know that transcription of these pro-regenerative genes remains significantly elevated for about a week after ES (Al-Majed et al., 2004). Additionally, if 10 min of ES is similar to 1 h of ES, could 5 min suffice?

#### Non-injury site ES therapies

Additionally, several studies induce ES on peripheral nerve targets to produce similar effects as Spinal Cord Stimulation (SCS), Dorsal Root Ganglion (DRG) or direct nerve stimulation to prevent substantial risks of very invasive procedures. Such can be divided into 4 broader classifications: Skeletal Muscle Stimulation (SMS), Percutaneous Electrical Stimulation, Subcutaneous Stimulation (SQS), and Transcutaneous Electrical Nerve Stimulation (TENS).

In SMS electrical pulses are delivered directly to the denervated muscle to evoke muscle contractions and indirectly stimulate the motor nerves. Muscle stimulation has shown some similar regeneration attributes as direct proximal nerve stimulation at injury site (Chu et al., 2022; Willand et al., 2016). SMS increases cytoplasmic free Ca^2+^ concentrations and upregulation of myogenic differentiation 1 (MYOD) by increased merging of adult stem cells with myofibers, promoting muscle fiber regeneration (Chu et al., 2022; Di Filippo et al., 2017). SMS also improves muscle regeneration capacity by reducing oxidative stress in muscle stem cells shown by lower superoxide production (Chu et al., 2022; Di Filippo et al., 2017). SMS induces metabolic and hypertrophic changes in muscle tissue with increased myotube size and activation of growth pathways (mTORC1 and ERK1/2; Chu et al., 2022; Khodabukus et al., 2018). SMS also leads to higher glucose uptake (Chu et al., 2022; Nedachi et al., 2008) and increased insulin activation, which can improve muscle endurance (Chu et al., 2022). By decreasing atrophy and priming the muscle for reinnervation, SMS contributes to functional recovery. Hence, the optimal time for SMS can be inferred as 3 days post-PNI (Chu et al., 2022).

Percutaneous ES and SQS offer procedures of shorter duration and opportunities to deliver multiple ES sessions without requiring additional surgeries (Hardy et al., 2024; Ni et al., 2023). The percutaneous ES protocol uses a needle or fine wire electrode that is inserted through a small skin incision, positioning it near the repaired nerve trunk without the need for an open surgical field. Subcutaneous ES uses a flexible cuff electrode that is tunneled under the skin. The cuff encircles the repaired nerve to allow for repeated stimulation sessions. Nerve stimulation in this case will allow for the injured nerves to become synchronously depolarized even though these electrodes are not placed directly on the nerve (Chu et al., 2022; Costello et al., 2023; Ni et al., 2023). SQS heavily relies on Electroacupuncture (EA) which can penetrate the skin, tissue, and skeletal muscle. It can have similar effects as TENS and SMS, but lacks effectiveness in width (Chu et al., 2022). Several studies indicate EA reducing apoptosis and inflammatory response post-PNI, while upregulating neurotrophic factors (Chu et al., 2022). However, recent studies focus on the damage caused by electrical overstimulation and use of threaded microneedle electrodes to deliver protective microspheres to reduce oxidative stress and cell damage (Liu et al., 2025).

TENS involves externally placing small adhesive electrode patches on the skin ~1–4 cm above or near the injured nerve trajectory. TENS is performed in the days to weeks following nerve repair to support early healing and to provide analgesia. TENS activates descending inhibitory pathways in the dorsal horn, which results in the release of endogenous opioids and in increase in gamma-aminobutyric acid (GABAergic) activity (Chen et al., 2024; Chu et al., 2022). TENS improves local circulation through vasodilation. This facilitates nutrient delivery, edema reduction, and inflammation (Chu et al., 2022; Costello et al., 2023; Hardy et al., 2024; Ni et al., 2023). TENS primarily target superficial sensory fibers for modulating sensory function and pain by blocking nociceptive signaling (Chu et al., 2022). Studies implementing ultra-high frequency (UHF) TENS also found decreased levels of pain-related neuropeptides and inflammatory indicators by modulating BDNF/MAPK pathways in DRG (Chen et al., 2024). However, SQS can be regarded as a better alternative than TENS to treat PNI because SQS affects large number of deep sensory fibers without dissipation from fat (Chu et al., 2022). TENS being an external ES strategy poses lower implementation risk as a non-invasive intervention.

Additionally, novel techniques like motor cortex stimulation (MCS) can be used to promote regeneration orthodromically through motor pathways (Nicolas et al., 2018). MCS could enhance the neuronal connections in CNS, in turn potentially improve motor coordination and functional recovery (Carmel et al., 2013; Nicolas et al., 2018). Acute MCS shows significant benefits in motor function recovery compared to chronic MCS and direct nerve stimulation (Nicolas et al., 2018). However, there are limited studies that discuss the potential underlying mechanisms in depth, specific effects on sensory aspects of PNI, and optimal parameters for clinical implementation of MCS in PNI.

### Scaffolds and conduits

Historically, nerve autografts have been the gold standard treatment for bridging long nerve gaps. This technique involves harvesting healthy nerve segments from another site in the body and transplanting them at the injury site (Gaudin et al., 2016; Grinsell and Keating, 2014; Hussain et al., 2020; Lopez-Leal and Court, 2016). However, autografts present significant drawbacks, including limited donor availability, neuroma formation, donor site morbidity, and the need for additional surgical procedures (Grinsell and Keating, 2014; Griffin et al., 2014; Moore et al., 2009). Synthetic and bioengineered nerve conduits have been developed to address these limitations while promoting nerve regeneration across longer gaps (Bryan et al., 2024; Dong X. et al., 2023; Wan et al., 2025; Zhao et al., 2024; Zhu et al., 2011).

Synthetic nerve guidance conduits were explored as an alternative to nerve autografting for the past 40 years (Zhu et al., 2011). Early designs relied on non-degradable silicone tubes which were immunogenic and prone to collapse (Merle et al., 1989). More recent conduit models utilize aligned microfiber and nanofiber scaffolds (Dong X. et al., 2023; Lopes et al., 2022; Yang et al., 2023; Zhu et al., 2011), with electrospun conduits mimicking the extracellular matrix (ECM) to enhance cellular adhesion and axonal guidance (Bryan et al., 2024; Wan et al., 2025). Advances in biomaterials have led to the incorporation of bioactive modifications, including growth factors and decellularized ECM to further support nerve regeneration (Bryan et al., 2024; Dong X. et al., 2023; Lopes et al., 2022). Additionally, multifunctional conduits with immunomodulatory properties and conductive scaffolds now enable electrical stimulation integration to accelerate the regenerative process (Bryan et al., 2024; Wan et al., 2025; Yang et al., 2023; Zhao et al., 2024; Zhu et al., 2011). In addition, strategies incorporating machine learning (ML) tools to optimize conduit architecture design and expedite fabrication are emerging in neuroengineering (Venkata Krishna and Ravi Sankar, 2023).

#### Scaffold materials selection

**Figure 5:**
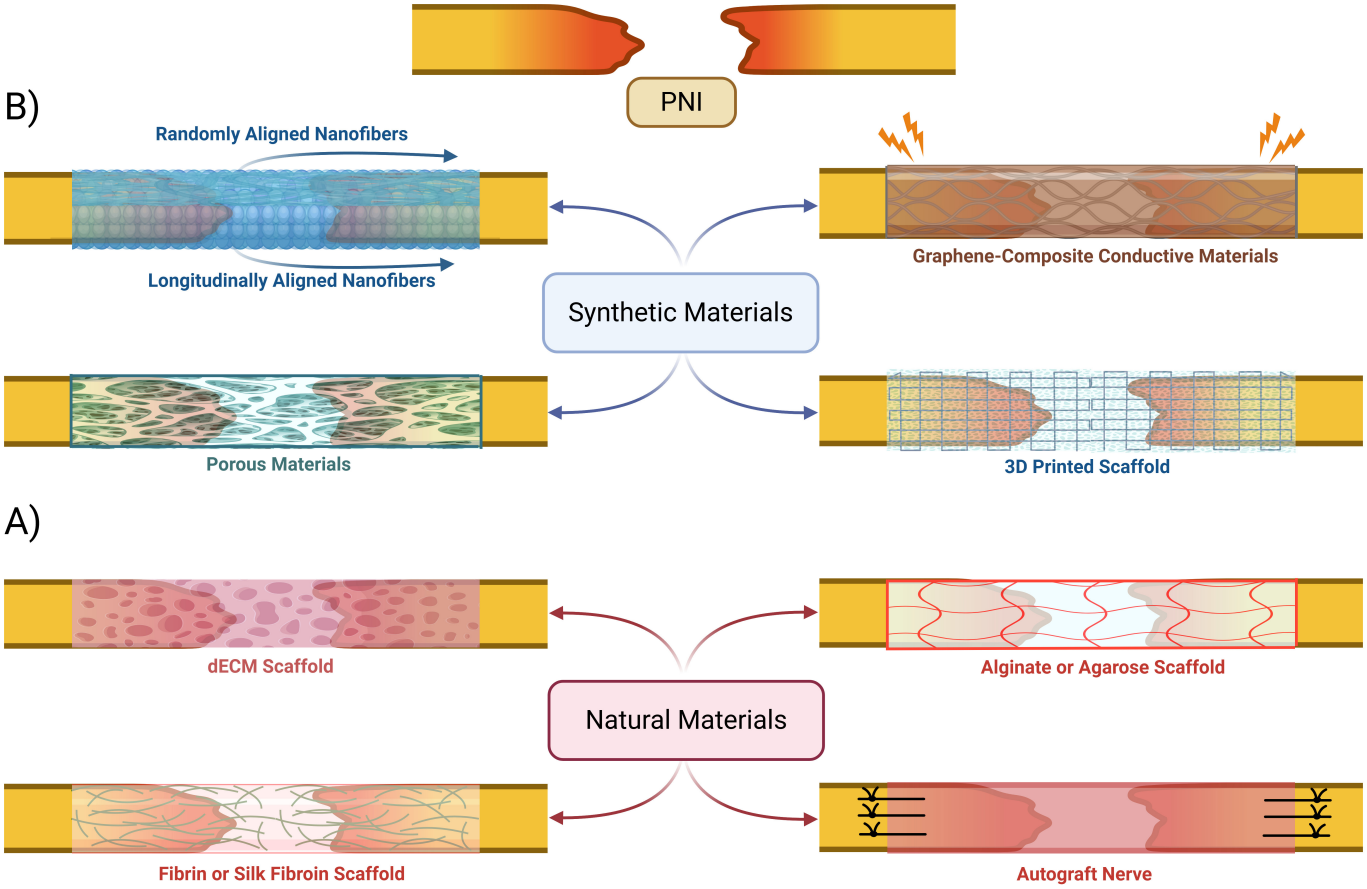
Bioengineered nerve conduits and scaffold-based gap repair strategies. An overview of various synthetic **(B)** and natural **(A)** materials that have been proposed to be used to create scaffolds and conduits to help bridge peripheral nerves in cases of injuries with gaps. dECM, decellularized extracellular matrix.

The selection of biomaterials for nerve scaffolds plays a crucial role in ensuring optimal mechanical and biological properties to support nerve regeneration. Several natural and synthetic biomaterials were used in the development of scaffolds and conduits (Figure 5) to facilitate neural outgrowth by targeting pro-regeneration factors (Dixon et al., 2018; Dong X. et al., 2023; Faroni et al., 2015). One of the primary challenges in scaffold design is matching the biomechanical properties of nerve tissue to mechanically support axonal regrowth (Grinsell and Keating, 2014; Venkata Krishna and Ravi Sankar, 2023; Wan et al., 2025).

##### Natural materials in bioscaffolds

Chitosan is a widely used natural biomaterial that is often combined with microfiber and nanofiber composites due to its excellent biocompatibility, biodegradability, and ability to support Schwann cell adhesion and proliferation (Scaccini et al., 2021; Venkata Krishna and Ravi Sankar, 2023; Zhang et al., 2021). Other natural biomaterials, including fibroin, keratin, dECM-based natural polymers and silk, have been explored for their strong biocompatibility and moderate mechanical integrity (Figure 5A; Hussain et al., 2020; Zhang et al., 2021). Despite their promise, neither of these biomaterials have demonstrated significant advantages over autografts in clinical applications (Zhu et al., 2011).

##### Synthetic and hybrid materials in bioscaffolds

Synthetic polymers such as poly(l-lactic acid; PLLA) and poly(l-lactic acid-co-ε-caprolactone; PLCL) have been developed to provide enhanced mechanical properties while maintaining a favorable microenvironment for nerve regeneration (Bryan et al., 2024; Dong X. et al., 2023). Randomly oriented fiber scaffolds offer structural support, but bi-layered nanofibrous conduits with longitudinally aligned inner layers and randomly oriented outer layers have been shown to improve axonal alignment and overall regeneration outcomes (Figure 5B; Lee et al., 2022; Wang et al., 2011; Zhu et al., 2011). An emerging strategy involves the use of synthetic piezoelectric polymers, such as poly(vinylidene fluoride; PVDF) and its copolymers with trifluoroethylene (PVDF-TrFE), which generate electrical signals in response to mechanical stress. When integrated with decellularized extracellular matrix (dECM), these materials create electroactive platforms that further enhance axonal regeneration by modulating cellular responses to microcurrents, potentially inducing nerve ES effects on regeneration (Bryan et al., 2024). Piezoelectrical biomaterials have shown potential for positive clinical impact in bone and cartilage regeneration (Liu et al., 2020, 2022, 2021), but clinical application is still limited due to toxicity and lack of biodegradability without further modifications to fabrication technique (Chorsi et al., 2019; Yang et al., 2023).

Other synthetic polymers, such as polycaprolactone (PCL), have been engineered into porous conduits for controlled release of insulin-like growth factor (IGF-1), which significantly enhances axonal growth *in vivo* (Wan et al., 2025). Recent advances have also introduced conductive biomaterials, including methacrylated gelatin combined with carbon nanotube sheets (CNT@GelMA) within a PLLA shell, to create self-powered scaffolds that leverage benefits associated with ES for nerve repair (Yang et al., 2023). Similarly, graphene-based scaffolds (GBSs) have been developed to enhance electrical conductivity (Figure 5B), promoting synergistic effects with electrical stimulation for more efficient nerve regeneration (Venkata Krishna and Ravi Sankar, 2023; Zhao et al., 2024). Hybrid natural-synthetic biomaterial approaches can potentially be very beneficial in clinical setting due to additional structural support combined with enhanced biochemical functionality (Dixon et al., 2018; Lee et al., 2022; Venkata Krishna and Ravi Sankar, 2023; Wan et al., 2025). For example, incorporating graphene or reduced graphene oxide (rGO) into PCL or PLLA scaffolds enhances electrical conductivity while maintaining mechanical stability (Bahremandi Tolou et al., 2021; Venkata Krishna and Ravi Sankar, 2023; Zhao et al., 2024). Similarly, chitosan-gelatin microcapsule composites allow for the fabrication of three-dimensional porous networks (Figure 5B) that enable sustained delivery of IGF-1 that supports long-term nerve regeneration (Wan et al., 2025). These combinatorial approaches illustrate the potential of next-generation scaffolds to surpass traditional autografts by providing both structural and biological advantages.

Modern approaches to nerve conduit fabrication involve 3D bioprinting, to enable patient-specific nerve guides (Figure 5B). This technique allows for precise control over scaffold architecture to facilitate the design of microchannel patterns that enhance axonal guidance (Dixon et al., 2018; Scaccini et al., 2021). Machine learning algorithms can be integrated to optimize conduit geometry, refine biomaterial composition, and tailor the doping levels of conductive fillers to improve both mechanical stability and electrical conductivity (Venkata Krishna and Ravi Sankar, 2023). There is significant potential for creating highly customized nerve repair solutions that are more efficient than traditional autografts.

#### Potential molecular targets in bioscaffold engineering

ECM-coated scaffolds that release bioactive substances such as IGF-1 bolster pro-repair SC activity (Wan et al., 2025). For instance, IGF-1-containing 3D hydrogels enhance neurite extension by affecting neurotrophic factor release and MAPK and PI3K pathway activation (Dinescu et al., 2025; Fu et al., 2021; Lee et al., 2022). Scaffolds that mimic the local ECM may stabilize the injury site and support regeneration at the tissue level (Bryan et al., 2024; Jain et al., 2019; Wan et al., 2025; Zhang et al., 2022). 3D ECM-based conduits provide micro-and nano-porous architectures that facilitate vascular ingrowth which ensures efficient Wallerian degeneration (Bryan et al., 2024; Dong X. et al., 2023; Wan et al., 2025; Yang et al., 2023; Zhao et al., 2024).

Electroconductive and graphene-based conduits combine the regenerative benefits of structural stability from conduits and biochemical cues from ES. dECM-enriched PVDF-TrFE scaffolds engage SC surface receptors to augment focal adhesion and reduce inflammatory signaling *in vitro* (Bryan et al., 2024). Scaffolds with conductive surfaces increase cell excitability by increasing calcium influx. This is accomplished by establishing mild electric fields in neurons from body movements (Liu et al., 2021; Yang et al., 2023; Bryan et al., 2024). Conductive hydrogels further optimize the local microenvironment by modulating immune responses. Scarring is minimized and pro-regenerative tissue remodeling is promoted through the regulation of cytokine release (Dong et al., 2021; Liu et al., 2021; Wan et al., 2025; Yang et al., 2023). Conductive and electroactive scaffolds enhance nerve regeneration by harnessing microcurrents to amplify neuronal membrane depolarization in regenerating growth cones. These scaffolds accelerate axonal bridging across nerve gaps (Bryan et al., 2024; Gaudin et al., 2016; Lee et al., 2022; Liu et al., 2021; Venkata Krishna and Ravi Sankar, 2023; Yang et al., 2023). Electrical signals, neurotrophic factor release, and immune cell infiltration can significantly accelerate axonal growth and promote reinnervation of distal muscles and sensory structures (Bryan et al., 2024; Gaudin et al., 2016; Tonazzini et al., 2017; Wan et al., 2025). When rGO or graphene-doped scaffolds are combined with ES, SCs exhibit increased myelin protein expression and produce more functional myelin sheaths (Zhao et al., 2024). These molecular and cellular interactions collectively contribute to a regenerative microenvironment that may accelerate nerve regeneration. The integration of bioactive scaffolds and electrical stimulation offers a multidisciplinary therapeutic approach.

In Nerve transection or large-gap PNI, the distal axon segments degenerate and form debris combined with severed myelin sheaths (Faroni et al., 2015; Hussain et al., 2020; Wan et al., 2025). Inadequate cellular debris clearance can prolong the regeneration process (Hussain et al., 2020; Wan et al., 2025). Early infiltration of macrophages and SCs accelerates axonal regeneration and debris clearance. This can be achieved through a scaffold design that incorporates aligned microfibers or nanofibers without compromising nascent axons (Dong et al., 2021; Dong X. et al., 2023; Wan et al., 2025).

The M1 to M2 macrophage transition is essential for the immediate post-injury myelin debris clearance, ECM remodeling, SC proliferation, and angiogenesis (Dong et al., 2021; Wan et al., 2025; Zhang et al., 2022). Rippled nanofiber-aligned conduits also influence SC adhesion, viability, short-term proliferation, and long-term growth (Masciullo et al., 2017). Aligned microfiber or nanofiber scaffolds promote SC migration along the longitudinal axis by supporting the M1 to M2 macrophage transition (Dong et al., 2021; Tonazzini et al., 2017). Anisotropic and longitudinally aligned micro-nanofibers may enhance functional recovery by preserving structural integrity and maintaining luminal patency. This creates an optimal microenvironment for effective regeneration and guided axonal outgrowth (Dixon et al., 2018; Wang et al., 2011; Yang et al., 2023; Zhu et al., 2011). Scaccini et al. (2021) further demonstrated that chitosan-based conduits with micro-grooved membranes promote enhanced cell migration and proliferation (scalene triangles) while facilitating directionally aligned cell growth (gratings). Aligned micro-nanofiber conduits are advantageous since they integrate with the surrounding tissue (Cao et al., 2010).

Widened nerve gaps resulting from the retraction of proximal and distal stumps following peripheral nerve injury (PNI) can further hinder the regenerative process and complicate functional recovery. Well-aligned conduits that maintain constant lumen patency offer a significant advantage over randomly oriented scaffolds which risk partial collapse or inconsistent bridging during the retraction phase (Dixon et al., 2018; Dong X. et al., 2023; Zhang et al., 2022). Over time, aligned conduits facilitate more efficient axonal growth across the gap. Another limitation posed by random-fiber scaffolds is the formation of thicker fibrotic capsules at 12 months (Zhu et al., 2011). These can restrict nerve enlargement and reduce the final nerve diameter. Aligned-fiber scaffolds, on the other hand, can promote the formation of a thinner, epineurium-like layer that is better at mimicking the natural nerve structure (Zhu et al., 2011). Axonal misdirection is a major barrier to successful nerve regeneration and reinnervation. A lack of topographical guidance could be addressed by minimizing sensory-motor mismatches that improve motor recovery (Cao et al., 2010; Dixon et al., 2018; Ferrari et al., 2010). Nanofibrous scaffolds can support clinically relevant conduction velocity and muscle function recovery comparable to autografts—an advantage not observed in random-fiber constructs (Dong X. et al., 2023; Pfister et al., 2007; Zhu et al., 2011). Scaffolds that lack native biomimicry can often induce excessive foreign-body interactions that lead to thick fibrotic tissue and persistent inflammation (Dong et al., 2021; Gaudin et al., 2016; Hussain et al., 2020).

## Future directions and conclusions

Diagnosing and treating PNI remains a challenge due to the intricate structure of peripheral nerves, the complex biomechanical nature of the nerve regeneration process, and the clinical variability in symptom presentation and functional recovery (Dong Y. et al., 2023; Silver et al., 2021). While direct epineurial suture repair and nerve transfers remain the gold standard for treating severe PNI where spontaneous recovery is unlikely, the high failure rate and the slow intrinsic rate of axonal growth highlight the need for strategies to enhance the nerve regeneration process. Bioengineered scaffolds, including natural, synthetic, and hybrid biomaterials, show significant promise in matching or even surpassing autograft efficacy. The integration of 3D bioprinting, decellularized ECM, and biomaterial optimization offers a promising avenue for a personalized medicine approach to PNI. Electrical stimulation has also emerged as a potential adjunct therapy for accelerating nerve regeneration. With future studies that definitively establish the efficacy and optimal parameters for these and other therapies to accelerate axonal regeneration, we can envision employing these strategies not only to restore function and peripheral nerve injuries, but other devastating neurological disorders, such as spinal cord injury, traumatic brain injury, and stroke, to name a few.
